# Phenomenological-control framing of suggestion-based interventions: a testable hypothesis for acceptability and implementation in psychiatric care

**DOI:** 10.3389/fpsyt.2026.1863780

**Published:** 2026-07-30

**Authors:** Adrian Sanger, Tadeusz Pietras

**Affiliations:** 1 Institute of Psychology, Polish Academy of Sciences, Warsaw, Poland; 2 Department of Clinical Pharmacology, Medical University of Lodz, Lodz, Poland

**Keywords:** hypnosis, phenomenological control, psychiatric care, suggestion-based interventions, treatment acceptability

## Abstract

This Hypothesis and Theory article draws on a targeted, non-systematic synthesis of relevant literature and does not report new empirical data. Suggestion-based interventions show clinical promise across several medical conditions, yet their uptake in routine care remains limited. Psychiatry may be a particularly informative context in which to examine framing-related barriers because treatment engagement, perceived agency, trust, acceptability, and appropriateness may be especially sensitive to how an intervention is described and understood. In response to these concerns, the article considers whether selected suggestion-based interventions could be presented through a phenomenological-control framing. Phenomenological control refers here to a patient-side capacity for the goal-directed regulation of subjective experience, whereas a phenomenological-control framing uses that capacity as the explanatory rationale for the intervention. The intervention itself remains a clinical procedure involving structured suggestions. The central hypothesis is that, relative to a hypnotic framing, a phenomenological-control framing may influence implementation-relevant responses without implying greater clinical efficacy. Patient-level predictions concern perceived agency, concerns about loss of control, acceptability, appropriateness, and willingness to engage. Provider-level predictions concern scientific credibility, appropriateness, and intentions to recommend, refer, adopt, or seek training. These predictions are conditional and may vary across individuals and contexts.

## 1 Introduction

A growing body of evidence supports the clinical usefulness of suggestion-based interventions, including hypnotic interventions, across medicine. In this article, suggestion-based interventions refer to clinical procedures in which explicit, structured suggestions constitute a central component intended to facilitate changes in subjective experience, perception, cognition, emotion, bodily sensation, behavior, or symptoms. The present argument focuses primarily on interventions conventionally described as hypnosis or hypnotherapy, as well as comparable procedures and clinical practices historically associated with hypnosis.

The APA Dictionary of Psychology defines hypnosis as “the procedure, or the state induced by that procedure, in which suggestion is used to evoke changes in sensation, perception, cognition, emotion, or control over motor behavior” (1). This definition reflects the fact that the term hypnosis is used to refer both to a clinical procedure and to a proposed state associated with that procedure. Because the nature of hypnosis and hypnotic suggestibility remain theoretically contested (2), the present article does not assume that a distinct hypnotic state is necessary to explain responses to suggestion. Instead, the term hypnosis is used operationally to refer to a clinical procedure and its associated context. Such procedures use structured suggestions, often accompanied by a hypnotic label, rationale, or induction, to facilitate intended changes in experience, behavior, or symptoms.

Among the most developed evidence bases are pain management, surgical and other medical procedures, and irritable bowel syndrome (3–5). An umbrella review of 49 meta-analyses published after 2000 encompassed 261 distinct primary studies. More than half of the reported effect estimates were medium or large, although the review also emphasized substantial methodological heterogeneity and variability in study quality (6).

The available psychiatric literature appears heterogeneous and, at present, more consistent with adjunctive than stand-alone use, particularly in anxiety-related conditions, depression, and post-traumatic stress disorder (PTSD) (7–10). The present article is not primarily concerned with whether psychiatric indications currently provide the strongest efficacy base for suggestion-based interventions. Rather, it focuses on psychiatry as a clinical context in which treatment engagement, perceived agency, trust, and treatment acceptability may be especially sensitive to how an intervention is conceptually framed and introduced. Psychiatric care may be a particularly informative setting in which to test such framing effects because treatment uptake often depends on how the intervention is understood in relation to autonomy, collaboration, and self-regulation. This makes psychiatry not merely another application area, but a relevant context in which implementation consequences of terminology may be especially visible.

Despite their clinical promise, suggestion-based interventions remain underused in routine practice (11). This implementation gap is unlikely to have a single cause and may reflect the strength and accessibility of the evidence base, training opportunities, professional norms, organizational resources, and the perceived fit of the intervention with existing clinical practice (12). One underexamined contributor to this gap may be that the word hypnosis evokes concerns about passivity, external control, manipulation, or reduced autonomy (13, 14), especially in settings where trust and collaboration are central to treatment uptake. Such framing may influence both clinicians’ perceptions of scientific credibility and appropriateness and patients’ expectations, concerns about loss of control, and willingness to engage. Potential mechanisms underlying these effects include expectancies, threat-related beliefs, and perceived loss of agency.

Hypnotherapy has been described as continuing to struggle with “image problems, mislabeling and stigmatization within psychiatry” (15, p. 388). In this respect, the implementation gap may depend not only on the evidence base or training availability, but also on the cultural meaning of the term hypnosis and on the explanatory models commonly used to present it. Terminology is therefore not proposed as a sufficient explanation of the implementation gap, but as one factor that may amplify or reduce existing barriers.

Within this context, the relevant question is not only whether a suggestion-based intervention produces clinical effects, but also how it is understood and evaluated by patients and clinicians. Clinical efficacy refers to changes in symptoms, functioning, or other health outcomes, whereas implementation denotes the uptake and integration of an intervention into routine clinical practice. Acceptability concerns the cognitive and emotional evaluation of an intervention by those delivering or receiving it. Appropriateness reflects its perceived fit, relevance, or compatibility for a particular setting, provider, or patient population (16, 17). Particularly relevant dimensions of acceptability include ethicality, intervention coherence, anticipated burden, and confidence in one’s ability to participate; treatment credibility is considered a related but distinct appraisal. Perceived agency refers here to the extent to which patients understand themselves as active participants in regulating their own experience rather than as passive recipients of an externally imposed procedure.

Accordingly, this article advances a testable implementation hypothesis: presenting selected suggestion-based interventions through a phenomenological-control rather than a hypnotic framing may influence implementation-relevant appraisals and behavioral intentions. The direction of these effects is not assumed to be uniform, because the label hypnosis may increase responsiveness for some individuals rather than function as a barrier (18). The relative effects of the two framings may therefore vary across individuals and contexts. The hypothesis concerns acceptability and uptake rather than improvements in symptom or functional outcomes.

## 2 Approach and scope of the literature

This article draws on a targeted, non-systematic synthesis of literature relevant to the development and evaluation of the proposed framing hypothesis. The searches and source selection were conducted by the first author. Relevant publications were identified primarily through searches of Google Scholar and PubMed, supplemented by backward and forward citation searching and AI-assisted literature-discovery tools. All included publications were read by the first author and assessed for relevance before inclusion. Searches were conducted primarily in English and were organized around the main components of the argument: the clinical efficacy of suggestion-based and hypnotic interventions, beliefs and misconceptions about hypnosis, implementation barriers, framing and expectancy effects, and the construct of phenomenological control.

The types of evidence prioritized differed across these domains. For clinical efficacy, preference was given to recent meta-analyses, umbrella reviews, and systematic reviews, particularly those published after 2010 when suitable evidence was available. Older publications were retained when they represented foundational conceptual or experimental contributions. Because evidence concerning implementation barriers, acceptability, and the prevalence of hypnosis-related misconceptions was more limited, relevant surveys, qualitative, experimental, and conceptual studies were also considered. Sources were selected on the basis of their relevance, recency, and contribution to the conceptual or empirical argument rather than through predefined systematic-review eligibility criteria. The synthesis was intended to support the development of a testable hypothesis, not to provide an exhaustive review or a formal assessment of the complete evidence base. No new empirical data were generated or analyzed for this article, and it therefore does not include a conventional Results section.

## 3 Implementation gap in suggestion-based interventions

Despite growing evidence of effectiveness in medicine, the implementation of hypnosis-based interventions remains limited. Consistent with the notion of an implementation gap, a nationwide survey of German anesthesia departments found that only 8.3% reported using hypnosis or relaxation procedures perioperatively, despite evidence supporting their efficacy (19). This estimate concerns routine perioperative care in general anesthesia departments rather than specialized hypnosis services or expert centers. Because the data come from anesthesiology, they provide indirect rather than direct evidence regarding implementation in psychiatric care.

Available studies suggest that this implementation gap is unlikely to have a single cause. In a qualitative study of hypnosis for chronic pain management, Gardner et al. (20) identified three major barriers: misconceptions about clinical hypnosis, reduced confidence in its use, and concerns regarding integration with existing treatment frameworks. These findings suggest that implementation barriers may involve both practical and organizational constraints and the way the intervention is understood.

Barriers may also operate at the patient and referral levels. Ganzevoort et al. (21) examined the perceptions of children, parents, and general practitioners regarding hypnotherapy for children with functional abdominal pain. They found that “societal constraints and considerations (e.g. stigma and cost) regarding hypnotherapy affected the willingness of children, parents, and GPs to use hypnotherapy.” Although this study was conducted in pediatric primary care rather than psychiatry, it is relevant because it links stigma and other contextual barriers with willingness to engage in or recommend treatment.

Implementation difficulties may persist even after clinicians receive relevant training. Providers have reported challenges transferring hypnosis-based methods into routine clinical settings, including uncertainty about integration with existing workflows, limited institutional support, and the need for further supervision or practical guidance (22, 23).

Most of this evidence comes from anesthesiology, pain management, pediatric care, or broader healthcare samples and should therefore be interpreted as indirect evidence for psychiatry. Nevertheless, it suggests that limited implementation may reflect several interacting barriers, including training and confidence, compatibility with existing treatment pathways, organizational constraints, cost, and beliefs about hypnosis. The following section examines more specifically whether hypnosis-related misconceptions, terminology, and explanatory framing may contribute to these barriers.

## 4 Hypnosis-related beliefs, terminology, and framing as potential implementation barriers

One potential contributor to these implementation barriers is the cultural and historical meaning associated with the word “hypnosis.” The sources of stigma surrounding the term can be traced to its roots in mesmerism and to popular cultural representations (14, 24). This issue is not new: Hull drew attention to it as early as 1929 (25). Popular representations may shape public perceptions of hypnosis and contribute to misconceptions that complicate efforts to present hypnotherapy as an evidence-based treatment (24, 26). For example, Singaporean television dramas portrayed hypnotherapy through problematic depictions of mental health professionals as manipulative, able to read minds, criminal, lacking compassion, or self-interested (24). Such portrayals may be relevant to help-seeking more broadly, as suggested by Vickery: “whether or not individuals choose to consult a health care professional has much to do with the public image of the condition and the profession” (27, p. 369).

Survey findings indicate that misconceptions are present across both patient and professional groups. In a survey of physicians, medical residents, family practice outpatients, and psychiatric outpatients, more than half of the patient participants endorsed misconceptions about hypnosis, and such beliefs were also present among some medical practitioners (28). A more recent Polish survey similarly found that misconceptions varied across participant groups. Psychotherapists using hypnosis and fifth-year psychology students held views most consistent with current scientific knowledge, whereas more misconceptions were reported among participants without prior exposure to hypnosis and those interested in paranormal phenomena (29). For a comprehensive overview of prevalent myths and misunderstandings, see Lynn et al. (13) and Geagea et al. (14).

Some studies have also examined the specific content of these beliefs. Desai et al. found that substantial proportions of surveyed health professionals incorrectly associated hypnosis with losing control, entering a state of sleep, and becoming dependent on the practitioner. As the authors concluded, “these myths may interfere in using hypnotherapy as [a] therapeutic tool” (30, p. 149).

The effects of these misconceptions may extend beyond public image. Nearly a century of research suggests that cultural beliefs about hypnosis can have real consequences for how people respond to hypnotic suggestions. Further discussion of these mechanisms is available in Barber (31), Council et al. (32), Baker and Kirsch (33). Relatedly, Lush et al. (34) noted findings consistent with the possibility that an explicitly hypnotic context may reduce responding among some individuals at the lower end of the distribution, perhaps because preconceptions about hypnosis encourage resistance or disengagement rather than cooperation. However, the effects of the hypnotic label are unlikely to be uniformly negative. In some contexts, explicitly labeling a procedure as hypnosis may increase responsiveness to suggestion, indicating that the label may sometimes facilitate rather than inhibit responding (18).

Professional attitudes may likewise depend partly on terminology. In a survey of anesthesia providers, 83% endorsed the use of positive suggestion in clinical practice, whereas only 42% supported hypnosis (35). Although the two terms may not have been understood as referring to fully identical procedures, this discrepancy is consistent with the possibility that terminology and explanatory framing influence professional acceptance.

These beliefs may also have implementation consequences for both practitioners and patients. Clinicians may be concerned that associations with pseudoscience could undermine their professional credibility. This concern may partly explain why hypnosis is not routinely integrated into mainstream training and clinical pathways (15, 28), while hypnosis-related constructs have remained weakly integrated with broader scientific frameworks (36, 37). Patients may express concerns about hypnotic interventions, including fears of losing control, coercion, or becoming stuck in hypnosis. Such concerns are documented in surveys of public beliefs and cultural attitudes toward hypnosis (e.g., (38)). In psychiatric and psychotherapeutic contexts, these beliefs may be especially consequential because fears of external influence or passivity may reduce perceived treatment acceptability.

Taken together, these findings do not establish that terminology is the primary cause of the implementation gap. They do, however, support examining hypnosis-related beliefs, labels, and explanatory framing as potentially modifiable contributors to professional acceptance, patient willingness to engage, and perceived compatibility with collaborative clinical care.

## 5 Psychiatric relevance of framing-related barriers

In psychiatric settings, the framing of an intervention may be especially important because treatment may depend not only on technical efficacy, but also on how the intervention is understood by the patient. Across psychiatric treatments, patient–clinician communication, treatment expectations, the therapeutic relationship, and treatment engagement have been examined as clinically relevant process factors (39, 40). Terms associated with passivity, external influence, or altered control may shape expectations before treatment even begins. This is particularly relevant to informed consent, where patients’ decisions may be influenced not only by the intervention itself, but also by the way it is described and by the patient’s understanding of their role in treatment (41, 42). Shared decision-making in psychiatric care is similarly grounded in a trusting relationship and is intended to support patient involvement and autonomy (43). In this context, the term “hypnosis” may evoke concerns about manipulation, loss of autonomy, or dependency, even when the actual procedure is collaborative.

Although these issues are not unique to psychiatry, they may be especially consequential in psychiatric and psychotherapeutic settings, where treatment often requires sustained collaboration and where concerns about autonomy or external influence may affect engagement, the therapeutic relationship, uptake, and adherence. Suggestion-based methods introduced as adjuncts to psychotherapy may be interpreted not simply as techniques, but also in terms of interpersonal influence and the patient’s role in producing therapeutic change. Expressions such as “being hypnotized” or being “susceptible to suggestions” may imply that the procedure is performed on the patient rather than carried out collaboratively with the patient. For some individuals, such language may reinforce expectations of passivity, external control, or reduced agency, which may in turn reduce perceived acceptability or willingness to engage.

For these reasons, the same framing-related barriers that appear in broader medical settings may have heightened significance in psychiatric care. Here, the way an intervention is named and explained may affect not only willingness to try it, but also the extent to which it is perceived as compatible with self-regulation, collaboration, and meaningful participation in treatment. This creates a rationale for testing whether a more agency-compatible and patient-centered framing of suggestion-based interventions influences perceived agency, treatment credibility, acceptability, and willingness to participate. It does not establish that such a framing will necessarily be more acceptable or that terminology alone determines clinical uptake.

## 6 Phenomenological control as a basis for a pragmatic clinical framing hypothesis

Concerns about terminology in hypnosis research are not new. From the late 1960s onward, Theodore X. Barber (44, 45), and later Nicholas Spanos (46), argued that “hypnosis” is a historically and culturally loaded label. In response, they advocated replacing the term with process-descriptive constructs such as “imaginative involvement” and “goal-directed fantasy.” In 2001, Kirsch and Braffman suggested that “hypnotizability is an inaccurate and misleading name for a very important trait, ability, or propensity that has little to do with the induction of hypnosis” (47, p. 58). These positions highlight that “hypnosis” can be understood as a historical and cultural descriptor for a family of practices and responses that rely on an underlying human ability. In this view, what is commonly described as hypnotic responding may be better understood as one expression of a broader patient-side capacity to shape subjective experience. Focusing on this underlying ability may help bypass misunderstandings and related barriers.

Using a more semantically neutral, capacity-focused framing to describe the intervention is not merely a matter of rebranding to avoid negative connotations. It may also address the issues described earlier, particularly those related to patient self-efficacy and agency. The criteria that such a pragmatic framing should satisfy include the following: (a) a focus on the patient-side capacity rather than the procedure or the provider, (b) semantic neutrality and minimal historical connotations, and (c) theory-agnostic scope sufficient to encompass the broader field of practice.

Terms such as “therapeutic suggestion” or “suggestion-based interventions” are more neutral than “hypnosis,” but they may still evoke an interpersonal model when they are presented primarily in terms of one person influencing another who is more or less susceptible to suggestion (48). In psychiatric settings, such a framing may reinforce concerns about passivity, external control, and reduced agency. Earlier alternatives such as “imaginative involvement” and “goal-directed fantasy” were historically important attempts to move beyond the culturally loaded term “hypnosis,” but they may be less suitable for the present purpose. Both terms foreground imagination and fantasy as if these were the core defining features of hypnotic responding, whereas later work has cast doubt on the view that suggestion-related imagery is either necessary or sufficient to explain responsiveness to suggestion (49). More recent reviews further suggest that hypnotic suggestion and voluntary imagery are not best understood as the same kind of process, which makes imagination-centered terminology too narrow for a broader clinical framing (50).

A potentially useful capacity-focused construct for this purpose is “phenomenological control,” introduced by Dienes, Lush, and their colleagues (48). Dienes and Lush define it as the capacity of individuals “to alter their subjective experience such that it misrepresents reality in ways consistent with their goals and such that the misrepresentation can be sustained over at least minutes despite clear contrary evidence” (51, p. 145). In the present article, phenomenological control refers to this underlying capacity. A phenomenological-control framing refers to explaining selected suggestion-based interventions in terms of that capacity, whereas the intervention itself remains a clinical procedure involving structured suggestions. In this framework, phenomenological control is not a substitute label for hypnosis, but a broader construct centered on a capacity for shaping subjective experience, of which hypnotic responding may be understood as one context-specific example. The term was introduced to avoid associations commonly carried by the word “hypnosis” (48). Importantly, the word “control” in this context refers to goal-directed regulation of subjective experience, not to suppression, coercion, or external domination.

The present proposal is not that a phenomenological-control framing has already been shown to be superior to a hypnotic framing, but that its implementation effects are empirically testable. Accordingly, the primary emphasis here is on the use of phenomenological control as a basis for clinical framing rather than on advancing a comprehensive re-theorization of hypnosis as a whole. This framing shifts the emphasis from a culturally loaded and procedure-centered label toward a more patient-centered description of the capacity to regulate subjective experience in a goal-directed way. In this respect, phenomenological control can be distinguished from “hypnosis” as a historically and culturally loaded label, from “suggestibility” as a term that may connote passive responsiveness to another person, and from “expectancy” as a construct referring more narrowly to anticipated outcomes. **Table 1** distinguishes phenomenological control as an underlying capacity, a phenomenological-control framing used to explain an intervention, and the clinical procedure presented through that framing.

**Table 1:** Distinction between phenomenological control, phenomenological-control framing, and a phenomenological-control-framed suggestion-based intervention.

Level	Meaning in the present article	Function	Illustrative patient-facing wording
Phenomenological control as a capacity	An individual capacity to shape subjective experience in a goal-directed manner. Responses may involve sensations, perceptions, emotions, or movements that subsequently feel spontaneous, effortless, or involuntary. Hypnotic responding may be understood as one context-specific expression of this broader capacity.	Identifies the patient-side capacity used to explain responses without requiring a distinct hypnotic state or context.	“People can sometimes engage with imagery and attention in ways that shape what they experience, even when the resulting sensation or movement feels as though it happens by itself.”
Phenomenological-control framing	An explanatory rationale that presents responses to a suggestion-based procedure in terms of the patient’s active, goal-directed engagement in shaping subjective experience. It acknowledges that the resulting response may feel involuntary without implying that it was externally imposed by the clinician.	Represents the proposed alternative to a framing centered on hypnosis, trance, susceptibility, or the clinician’s control over the patient. It is hypothesized to influence perceived agency, treatment credibility, acceptability, appropriateness, and willingness to engage.	“This method may use imagery and focused attention to explore how you can shape aspects of your experience. You remain an active participant, although some responses may feel spontaneous or as if they happen on their own.”
Phenomenological-control-framed suggestion-based intervention	A clinical procedure involving structured suggestions and, where relevant, imagery, attentional focus, verbal guidance, or related instructions that is introduced and explained using a phenomenological- control rationale.	Allows the effects of explanatory framing to be tested while the core clinical procedure is held constant. It therefore permits comparison with the same or a closely matched procedure presented through explicitly hypnotic terminology and explanation.	“During the exercise, I will guide you to focus on particular images, sensations, or experiences. Your role is to engage with them as fully as you can. Any resulting changes may feel effortless or automatic, but you remain actively involved and can pause or stop the exercise at any time.”

The patient-facing examples are illustrative rather than standardized scripts. They are intended to demonstrate the conceptual distinctions in the table and would require adaptation to the clinical procedure, population, and context. They are not substitutes for transparent informed consent and should not be used to obscure the nature of the procedure or its relationship to practices conventionally described as hypnosis when that information is relevant to the patient’s decision to participate.

In psychiatric contexts, this framing may be clinically useful because it can present the intervention as a form of active experiential regulation rather than as something primarily done to the patient. Whether such framing reduces associations with loss of control and external influence, and thereby improves acceptability or engagement, remains an empirical question.

## 7 Proposed framing model

The present hypothesis can be summarized as a model in which the framing used to introduce a suggestion-based intervention is hypothesized to influence how that intervention is initially understood and evaluated. A hypnotic framing and a phenomenological-control framing may evoke different expectations and appraisals concerning external control, personal involvement, scientific credibility, threat, and the patient’s role in producing the response. These appraisals may, in turn, influence proximal implementation-relevant outcomes, including perceived agency, treatment credibility, acceptability, and appropriateness. These proximal outcomes may then influence behavioral intentions, including willingness to engage with, recommend, refer for, or adopt the intervention. The model does not assume that framing directly changes the clinical efficacy of the underlying procedure.

The model is explicitly conditional rather than universally directional. Any effects of framing may depend on prior experience with hypnosis, baseline beliefs and misconceptions, cultural context, clinical population, and professional background. For individuals who associate hypnosis with credibility, therapeutic potency, or positive expectations, an explicitly hypnotic framing may be more acceptable or engaging than an alternative framing. Conversely, individuals who associate hypnosis with external control, manipulation, passivity, or pseudoscience may respond more favorably to a phenomenological-control framing. The hypothesis therefore predicts variation in framing effects across individuals and contexts rather than a uniform advantage of either terminology.

The proposed pathways may differ between patients and clinicians. At the patient level, framing may influence perceived agency, concerns about loss of control, treatment credibility, acceptability, and willingness to begin or continue the intervention. At the provider level, framing may influence perceived scientific credibility, appropriateness, compatibility with existing clinical models, and intentions to recommend, refer for, adopt, or seek training in the intervention. These variables should be treated as related but distinct: a framing may, for example, increase perceived agency without increasing credibility, or improve provider appropriateness ratings without affecting patient willingness to engage.

**Figure 1:**
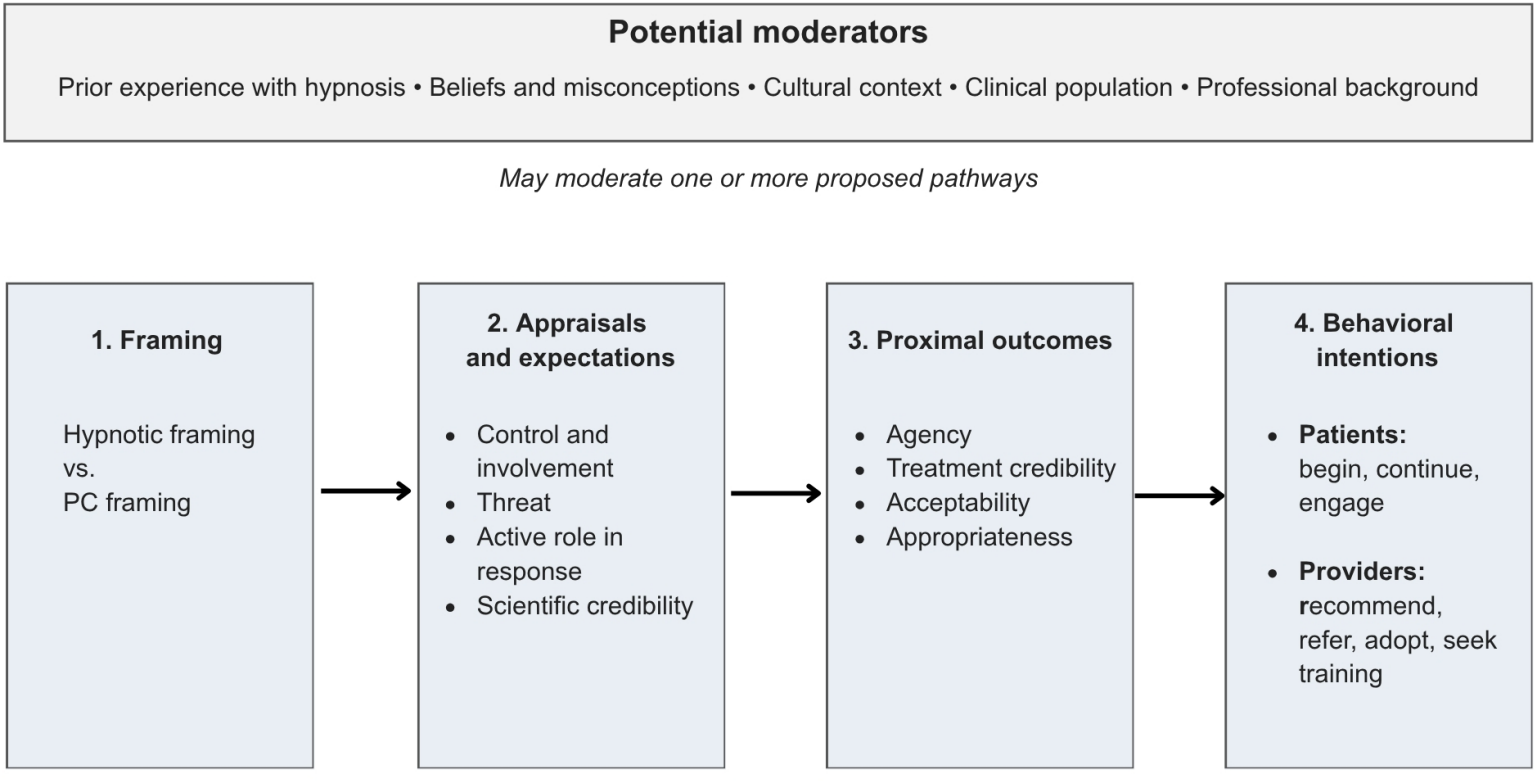
Hypothesized pathways through which clinical framing may influence implementation-related responses. The proposed pathways may vary across individuals and contexts. PC denotes phenomenological control.

This model is proposed as a testable account of how explanatory framing may influence implementation-related responses, rather than as an established mechanism or a recommendation to replace hypnotic terminology in clinical practice. Its central prediction is that holding the core procedure constant while varying its framing may produce differences in appraisals, perceived agency, credibility, acceptability, appropriateness, and behavioral intentions. These effects may differ between patients and clinicians and may be moderated by prior beliefs, experience, and context. The model does not require framing to alter the direct clinical effects of the procedure, and each proposed pathway can be examined separately. The hypothesized relationships are summarized in **Figure 1**.

## 8 Discussion

### 8.1 Clinical implications

In clinical practice, this reframing would involve presenting suggestion-based interventions as procedures that draw on patients’ capacity for active, goal-directed regulation of subjective experience (51), rather than as procedures performed on passive recipients. In psychiatry and psychotherapy, such a shift may be especially relevant because it aligns the intervention with agency, self-regulation, and collaborative treatment models rather than passivity or external control. Whether this facilitates more acceptable and explicit integration of suggestion-based methods into existing therapeutic pathways remains an empirical question. Such integration may be particularly relevant in areas where the psychiatric literature is suggestive but methodologically uneven, including anxiety-related conditions, PTSD, and depression (7, 9, 10). Broader reviews also identify preliminary evidence concerning sleep disorders (52).

More broadly, this framing may help clinicians present suggestion-based methods in a way that supports patients’ active involvement in regulating experience within clinical contexts. Because “phenomenological control” may be too technical for routine patient-facing communication, the scientific construct need not be used verbatim when introducing the intervention. At the same time, in professional or research contexts, a technical and semantically neutral term could increase perceived scientific credibility for some audiences, although this possibility also requires empirical testing. In patient-facing communication, clinicians could instead use a plain-language explanation that emphasizes the patient’s active role while acknowledging that some responses may feel spontaneous or involuntary. For example, when a procedure involves imagery and focused attention, rather than saying “Today I am going to hypnotize you,” a clinician might explain: “Today we will use structured verbal guidance, imagery, and focused attention to explore how you can shape aspects of your experience. You will remain actively involved, although some responses may feel spontaneous or as if they happen on their own.”

The intervention may therefore be framed as a method that draws on the patient’s capacity for experiential self-regulation, with possible benefits for self-efficacy, engagement, and treatment acceptability. Importantly, this shift does not imply the formation of a new specialty, the introduction of universal psychology training, or a move away from pharmacological approaches. More broadly, suggestion-based principles may also be relevant to routine clinical communication. As Häuser et al. noted, waking suggestions may form part of effective doctor–patient communication in everyday clinical situations (52).

Any use of alternative framing should remain consistent with transparent informed consent. A phenomenological-control framing should not obscure the actual components of the procedure or be used simply to avoid disclosing its relationship to practices conventionally described as hypnosis. Patients should receive a comprehensible explanation of the intervention, including its use of structured suggestions and any relevant use of verbal guidance, imagery, attentional focus, or other procedural elements. They should also be informed about its intended purpose, available alternatives, and their right to ask questions, decline, pause, or discontinue the procedure. The aim of reframing should therefore be to improve conceptual clarity and support autonomy, not to conceal information that may be relevant to the patient’s decision to participate.

### 8.2 Research implications

A similar reframing may also have implications for research on suggestion-based interventions. If the central issue is not only the procedure itself but also how it is conceptually framed, future studies may benefit from examining the role of patient-side capacities alongside implementation-relevant variables such as perceived agency, treatment credibility, acceptability, appropriateness, and willingness to engage. In psychiatric contexts, this may be especially important because uptake and adherence may be shaped in part by how the intervention is understood in relation to autonomy, collaboration, and self-regulation. Accordingly, research should distinguish implementation-relevant outcomes from clinical outcomes and examine whether different framings affect the expectations, evaluations, and behavioral intentions of both patients and clinicians before and during treatment. Such studies should not assume that framing-related differences in acceptability or willingness to engage will necessarily translate into changes in symptoms or functioning.

### 8.3 Testable hypotheses

To advance this framework, we propose two distinct framing-related hypotheses: one at the provider level and a second at the patient level.

At the provider level, we hypothesize that clinicians will demonstrate greater readiness to refer patients for treatment and greater willingness to participate in related training when the intervention is presented using a phenomenological-control framing rather than a hypnotic framing. To test this hypothesis, future research could use a randomized vignette experiment based on the logic of Stone et al. (35). Clinicians would be randomly assigned to otherwise identical descriptions of an intervention that differ only in their labeling and explanatory rationale. Outcomes could include readiness to refer, willingness to seek training, perceived credibility, perceived appropriateness, compatibility with existing treatments, and adoption intentions. Moderators such as prior exposure to hypnosis, professional background, and baseline attitudes or skepticism should also be examined.

At the patient level, we hypothesize that patients will report lower levels of anxiety regarding loss of control and a greater sense of agency when the method is presented using a phenomenological-control framing rather than a hypnotic framing. For an initial proof-of-concept randomized framing trial, a relatively homogeneous sample of adult outpatients with an anxiety-related disorder or depression could be recruited. Participants would be randomly assigned to receive different introductions to the same core clinical procedure. The procedure itself would be held constant across conditions, while only the intervention label and explanatory rationale would vary. The method could be introduced as an adjunct to standard psychiatric care, including pharmacotherapy. Eligibility should be based on the capacity to provide informed consent, sufficient comprehension to complete the study procedures, and the absence of an acute psychiatric presentation requiring immediate treatment or inpatient care, rather than on diagnosis alone. Enrollment could be postponed when current symptoms—such as active psychosis, severe persecutory ideation, or severe dissociation—would interfere with informed consent, comprehension of the framing manipulation, or safe participation. Subsequent studies could examine these populations using specialist protocols and enhanced monitoring.

Possible co-primary outcomes would be perceived agency and anxiety regarding loss of control. Secondary outcomes could include treatment credibility, acceptability, perceived appropriateness, and willingness to begin or continue the intervention. Symptom and functional outcomes could be included as exploratory measures, but an initial framing trial should not be interpreted as a test of the clinical efficacy of the underlying procedure.

Safety monitoring should include prospective assessment of adverse events, acute distress, symptom exacerbation, and dissociative reactions during and after the study procedure. Participants should be reminded that they may pause or stop the procedure or discontinue their participation at any time. Prespecified procedures should also be available for clinical review, follow-up, or referral if concerning reactions occur.

Both provider- and patient-level studies should include a comprehension check and manipulation checks following presentation of the rationale. Participants could rate the extent to which they perceived the intervention as externally controlled or collaborative, active or passive, scientifically credible, threatening, and similar to hypnosis. These measures would help determine whether the framing manipulation changed the intended appraisals rather than merely altering the label.

These hypotheses are not intended as direct claims that reframing alone would improve clinical outcomes. Rather, they are intended to test whether conceptual framing may influence implementation-relevant variables such as acceptability, perceived agency, appropriateness, and willingness to engage. Future framing trials should follow best-practice recommendations for clinical hypnosis research, including clear reporting of participant education, the exact intervention label and rationale, induction and procedural content, prior experience with hypnosis, expectancies, proposed mechanism variables, and adverse events, as well as provision of intervention materials where possible (53).

### 8.4 Limitations

Several limitations of the present argument should be acknowledged. First, although suggestion-based interventions show promise in psychiatric contexts, the psychiatric evidence base remains less robust than in pain, medical procedures, and irritable bowel syndrome, and is marked by substantial heterogeneity in populations, interventions, and outcome measures.

Second, using a phenomenological-control framing should not be understood as a substitute for stronger randomized trials or as evidence that reframing alone improves clinical outcomes. Rather, the present proposal is that conceptual framing may influence acceptability, engagement, and implementation-related variables, which themselves warrant empirical testing. It is also possible that such reframing could alter acceptability without improving symptom or functional outcomes.

Third, the effects of this framing may vary across individuals and across clinical and cultural contexts, and the proposed reframing may introduce new ambiguities or communication challenges. In particular, the term phenomenological control may prove too technical for routine patient-facing use in some settings. Relatedly, the proposed shift may be perceived by some clinicians or patients as cosmetic rebranding rather than as a substantively different and more useful way of conceptualizing the intervention.

Fourth, the term hypnosis may not always function as a barrier. In some contexts, it may enhance rather than reduce positive expectancies, credibility, or willingness to engage. Reframing may therefore produce beneficial, unfavorable, or null effects depending on the individual and context. Using a more neutral framing could in some cases weaken rather than strengthen treatment uptake. For this reason, the present proposal should not be interpreted as a recommendation for immediate adoption or as a universal recommendation to abandon the term hypnosis, but rather as a testable hypothesis about how alternative framing may function under specific clinical conditions.

### 8.5 Conclusion

The available evidence suggests that suggestion-based interventions face an implementation gap in medicine, and psychiatry may be a particularly important context in which to examine it because agency, trust, and treatment acceptability are often central to uptake and adherence. One plausible contributor to this gap is the culturally loaded conceptual framing associated with hypnosis. In the present article, phenomenological control refers to a patient-side capacity for the goal-directed alteration of subjective experience. Framing selected suggestion-based interventions in terms of this capacity is proposed not as a proven replacement for hypnotic terminology or framing, but as a pragmatic and testable implementation hypothesis that may, under specific conditions, be perceived as more compatible with patient-centered, collaborative, and agency-compatible models of psychiatric care.

## Data Availability

No datasets were generated or analyzed for this study.

## References

[1] American Psychological Association . Hypnosis. In: APA Dictionary of Psychology. Washington, DC: American Psychological Association (2018). Available online at: https://dictionary.apa.org/hypnosis (Accessed July 2, 2026).

[2] Geagea D Ogez D Kimble R Tyack Z . Redefining hypnosis: A narrative review of theories to move towards an integrative model. Complement Ther Clin Pract. (2024) 54:101826. doi: 10.1016/j.ctcp.2023.101826 38199053

[3] Milling LS Valentine KE LoStimolo LM Nett AM McCarley HS . Hypnosis and the alleviation of clinical pain: A comprehensive meta-analysis. Int J Clin Exp Hypn. (2021) 69:297–322. doi: 10.1080/00207144.2021.1920330 34038322

[4] Tefikow S Barth J Maichrowitz S Beelmann A Strauss B Rosendahl J . Efficacy of hypnosis in adults undergoing surgery or medical procedures: A meta-analysis of randomized controlled trials. Clin Psychol Rev. (2013) 33:623–36. doi: 10.1016/j.cpr.2013.03.005 23628907

[5] Adler EC Levine EH Ibarra AN Boparai ES Hung Y McCrary QD . Gut‐directed hypnotherapy for irritable bowel syndrome: A systematic review and meta‐analysis. Neurogastroenterol Motil. (2025) 37:e70037. doi: 10.1111/nmo.70037 40179285

[6] Rosendahl J Alldredge CT Haddenhorst A . Meta-analytic evidence on the efficacy of hypnosis for mental and somatic health issues: A 20-year perspective. Front Psychol. (2024) 14:1330238. doi: 10.3389/fpsyg.2023.1330238 38268815 PMC10807512

[7] O’Toole SK Solomon SL Bergdahl SA . A meta‐analysis of hypnotherapeutic techniques in the treatment of PTSD symptoms. J Trauma Stress. (2016) 29:97–100. doi: 10.1002/jts.22077 26855228

[8] Chan NA Zhang Z Yin G Li Z Ho RC . Update on hypnotherapy for psychiatrists. BJPsych Adv. (2023) 29:381–7. doi: 10.1192/bja.2021.54

[9] Souza FL Moura MS Vieira Almeida Silva J Elkins G . Hypnosis for depression: Systematic review of randomized clinical trials with meta-analysis. Complement Ther Clin Pract. (2024) 57:101913. doi: 10.1016/j.ctcp.2024.101913 39388785

[10] Valentine KE Milling LS Clark LJ Moriarty CL . The efficacy of hypnosis as a treatment for anxiety: A meta-analysis. Int J Clin Exp Hypn. (2019) 67:336–63. doi: 10.1080/00207144.2019.1613863 31251710

[11] Yeh VM Schnur JB Montgomery GH . Disseminating hypnosis to health care settings: Applying the RE-AIM framework. Psychol Conscious Theory Res Pract. (2014) 1:213–28. doi: 10.1037/cns0000012 25267941 PMC4175906

[12] Damschroder LJ Reardon CM Widerquist MAO Lowery J . The updated Consolidated Framework for Implementation Research based on user feedback. Implement Sci. (2022) 17:75. doi: 10.1186/s13012-022-01245-0 36309746 PMC9617234

[13] Lynn SJ Kirsch I Terhune DB Green JP . Myths and misconceptions about hypnosis and suggestion: Separating fact and fiction. Appl Cognit Psychol. (2020) 34:1253–64. doi: 10.1002/acp.3730 41531421

[14] Geagea D Ogez D Kimble R Tyack Z . Demystifying hypnosis: Unravelling facts, exploring the historical roots of myths, and discerning what is hypnosis. Complement Ther Clin Pract. (2023) 52:101776. doi: 10.1016/j.ctcp.2023.101776 37402329

[15] Orewa H Udo I . Hypnotherapy and therapeutic suggestion: Bridging the gap between evidence and utility. BJPsych Adv. (2023) 29:388–90. doi: 10.1192/bja.2022.68

[16] Sekhon M Cartwright M Francis JJ . Acceptability of healthcare interventions: An overview of reviews and development of a theoretical framework. BMC Health Serv Res. (2017) 17:88. doi: 10.1186/s12913-017-2031-8 28126032 PMC5267473

[17] Proctor E Silmere H Raghavan R Hovmand P Aarons G Bunger A . Outcomes for implementation research: Conceptual distinctions, measurement challenges, and research agenda. Adm Policy Ment Health. (2011) 38:65–76. doi: 10.1007/s10488-010-0319-7 20957426 PMC3068522

[18] Gandhi B Oakley DA . Does “hypnosis” by any other name smell as sweet? The efficacy of “hypnotic” inductions depends on the label “hypnosis”. Conscious Cognit. (2005) 14:304–15. doi: 10.1016/j.concog.2004.12.004 15950884

[19] Bügers L Wähner A Schubert AK Dinges HC Torossian A Volberg C . Hypnosis support in anaesthesia is rarely used in German anaesthesia departments - a nationwide survey among leading physicians of anaesthesia departments. BMC Anesthesiol. (2024) 24:314. doi: 10.1186/s12871-024-02705-4 39242504 PMC11378643

[20] Gardner T O’Hagan E Gilanyi YL McAuley JH Jensen MP Rizzo RR . Using hypnosis in clinical practice for the management of chronic pain: A qualitative study. Patient Educ Couns. (2024) 119:108097. doi: 10.1016/j.pec.2023.108097 38065021

[21] Ganzevoort IN van der Veen AL Alma MA Karg A Berger MY Holtman GA . Perceptions of hypnotherapy for children with functional abdominal pain: A qualitative study. Fam Pract. (2025) 42:cmaf066. doi: 10.1093/fampra/cmaf066 40910517 PMC12411915

[22] McKernan LC Finn MTM Patterson DR Williams RM Jensen MP . Clinical hypnosis for chronic pain in outpatient integrative medicine: An implementation and training model. J Altern Complement Med. (2020) 26:107–12. doi: 10.1089/acm.2019.0259 31904997 PMC7044758

[23] Carney LM Schnur JB Morgan O Green JP Montgomery GH . Enhancing hypnosis training to promote transfer to clinical practice for cancer pain management: A qualitative analysis of providers’ perceived needs. Int J Clin Exp Hypn. (2025) 73:141–55. doi: 10.1080/00207144.2025.2481901 40179354 PMC12054595

[24] Matthews G Ho M . Mental health treatments and the influence of culture: Portrayals of hypnotherapy and electroconvulsive therapy in Singaporean television dramas. Med Humanit. (2025) 51:13–25. doi: 10.1136/medhum-2023-012854 38991757

[25] Hull CL . Hypnotism in scientific perspective. Sci Mon. (1929) 29:154–62.

[26] Ardian J . Indonesian society’s perception of hypnotherapy compared to other types of psychotherapy: A potential and challenge. J Psychiatry Psychol Behav Res. (2022) 3:1–6. doi: 10.21776/ub.jppbr.2022.003.02.1

[27] Vickery K . Widening the psychiatric gaze: Reflections on PsychoDoctor, depression, and recent transitions in Japanese mental health care. Transcult Psychiatry. (2010) 47:363–91. doi: 10.1177/1363461510375162 20688796

[28] Elkins GR Wall VJ . Medical referrals for hypnotherapy: Opinions of physicians, residents, family practice outpatients, and psychiatry outpatients. Am J Clin Hypn. (1996) 38:254–62. doi: 10.1080/00029157.1996.10403349 8799033

[29] Baster J Polak M Szpitalak M Dudek I Polczyk R . Survey of beliefs about hypnosis among students, therapists, followers of paranormal beliefs, and the general public in Poland. Int J Clin Exp Hypn. (2023) 71:350–7. doi: 10.1080/00207144.2023.2251567 37682078

[30] Desai G Chaturvedi SK Ramachandra S . Hypnotherapy: Fact or fiction: A review in palliative care and opinions of health professionals. Indian J Palliat Care. (2011) 17:146–9. doi: 10.4103/0973-1075.84537 21976856 PMC3183605

[31] Barber TX . Measuring “hypnotic-like” suggestibility with and without “hypnotic induction”; Psychometric properties, norms, and variables influencing response to the Barber Suggestibility Scale (BSS). Psychol Rep. (1965) 16:809–44. doi: 10.2466/pr0.1965.16.3.809 14303013

[32] Council JR Kirsch I Vickery AR Carlson D . “ 'Trance' versus 'skill' hypnotic inductions: The effects of credibility, expectancy, and experimenter modeling”. J Consult Clin Psychol. (1983) 51:432–40. doi: 10.1037/0022-006X.51.3.432 6863705

[33] Baker SL Kirsch I . Hypnotic and placebo analgesia: Order effects and the placebo label. Contemp Hypn. (1993) 10:117–26.

[34] Lush P Scott RB Seth AK Dienes Z . The phenomenological control scale: Measuring the capacity for creating illusory nonvolition, hallucination and delusion. Collabra Psychol. (2021) 7:29542. doi: 10.1525/collabra.29542 33021500

[35] Stone AB Sheinberg R Bertram A Seymour AR . Are anesthesia providers ready for hypnosis? Anesthesia providers’ attitudes toward hypnotherapy. Am J Clin Hypn. (2016) 58:411–8. doi: 10.1080/00029157.2015.1136589 27003489

[36] Nadon R . What this field needs is a good nomological network. Int J Clin Exp Hypn. (1997) 45:314–23. doi: 10.1080/00207149708416132 9204643

[37] Dell PF . Reconsidering the autohypnotic model of the dissociative disorders. J Trauma Dissociation. (2019) 20:48–78. doi: 10.1080/15299732.2018.1451806 29565750

[38] Green JP Page RA Rasekhy R Johnson LK Bernhardt SE . Cultural views and attitudes about hypnosis: A survey of college students across four countries. Int J Clin Exp Hypn. (2006) 54:263–80. doi: 10.1080/00207140600689439 16766439

[39] Priebe S Conneely M McCabe R Bird V . What can clinicians do to improve outcomes across psychiatric treatments: A conceptual review of non-specific components. Epidemiol Psychiatr Sci. (2019) 29:e48. doi: 10.1017/S2045796019000428 31412975 PMC8061300

[40] Bourke E Barker C Fornells-Ambrojo M . Systematic review and meta-analysis of therapeutic alliance, engagement, and outcome in psychological therapies for psychosis. Psychol Psychother. (2021) 94:822–53. doi: 10.1111/papt.12330 33569885

[41] Fisher CB Oransky M . Informed consent to psychotherapy: Protecting the dignity and respecting the autonomy of patients. J Clin Psychol. (2008) 64:576–88. doi: 10.1002/jclp.20472 18381749

[42] Gerke L Pauls F Ladwig S Liebherz S Reininger KM Kriston L . Optimizing treatment expectations and decision making through informed consent for psychotherapy: A randomized controlled trial. J Consult Clin Psychol. (2024) 92:93–104. doi: 10.1037/ccp0000851 37971812

[43] Gurtner C Schols JMGA Lohrmann C Halfens RJG Hahn S . Conceptual understanding and applicability of shared decision-making in psychiatric care: An integrative review. J Psychiatr Ment Health Nurs. (2021) 28:531–48. doi: 10.1111/jpm.12712 33191536

[44] Barber TX . Hypnosis: A Scientific Approach. New York, NY: Van Nostrand Reinhold (1969).

[45] Barber TX Spanos NP Chaves JF . Hypnosis, Imagination and Human Potentialities. New York, NY: Pergamon Press (1974).

[46] Spanos NP . Hypnotic behavior: A social-psychological interpretation of amnesia, analgesia, and “trance logic”. Behav Brain Sci. (1986) 9:449–67. doi: 10.1017/S0140525X00046537 41292463

[47] Kirsch I Braffman W . Imaginative suggestibility and hypnotizability. Curr Dir Psychol Sci. (2001) 10:57–61. doi: 10.1111/1467-8721.00115 10510510

[48] Dienes Z Lush P Palfi B Roseboom W Scott R Parris B . Phenomenological control as cold control. Psychol Conscious Theory Res Pract. (2022) 9:101–16. doi: 10.1037/cns0000230 27371692

[49] Lynn SJ Snodgrass M Rhue JW Hardaway R . Goal-directed fantasy, hypnotic susceptibility, and expectancies. J Pers Soc Psychol. (1987) 53:933–8. doi: 10.1037/0022-3514.53.5.933 3681658

[50] Terhune DB Oakley DA . Hypnosis and imagination. In: Abraham A , editor. The Cambridge Handbook of the Imagination. Cambridge: Cambridge University Press (2020) 711–27. doi: 10.1017/9781108580298.043

[51] Dienes Z Lush P . The role of phenomenological control in experience. Curr Dir Psychol Sci. (2023) 32:145–51. doi: 10.1177/09637214221150521

[52] Häuser W Hagl M Schmierer A Hansen E . The efficacy, safety and applications of medical hypnosis. Dtsch Arzteblatt Int. (2016) 113:289–96. doi: 10.3238/arztebl.2016.0289 27173407 PMC4873672

[53] Kekecs Z Moss D Whorwell PJ Varga K Terhune DB Shenefelt PD . Best practice recommendations for conducting and reporting controlled trials in clinical hypnosis research. J Evid Based Integr Med. (2024) 29:2515690X241274538. doi: 10.1177/2515690X241274538 39403729 PMC11483803

[54] Sanger A Pietras T . Phenomenological-control framing of suggestion-based interventions: a testable hypothesis for acceptability and implementation in psychiatric care. PsyArXiv. (2026). doi: 10.31234/osf.io/h4gw5_v3 PMC13468807 42597346

